# Healthcare System Impact on Deceased Organ Donation and Transplantation: A Comparison Between the Top 10 Organ Donor Countries With 4 Countries in Southeast Asia

**DOI:** 10.3389/ti.2023.11233

**Published:** 2023-08-30

**Authors:** Sandra Cowie, Seow-Huey Choy, Diana Mohd Shah, Maria Paula Gomez, Boon-Koon Yoong, Jun-Kit Koong

**Affiliations:** ^1^ Department of Epidemiology, Biostatistics and Occupational Health, McGill University, Montreal, QC, Canada; ^2^ Faculty of Medicine, University of Malaya, Kuala Lumpur, Malaysia; ^3^ National Transplant Resource Centre, Kuala Lumpur, Malaysia; ^4^ Donation and Transplant Institute, Barcelona, Spain

**Keywords:** transplantation, organ donation, deceased donation, Southeast Asia (SEA), healthcare systems

## Abstract

The need for organ donation is constantly increasing. Some countries have made improvements, while others, such as countries in Southeast Asia (SEA), have some of the lowest rates of deceased donors (pmp). This review aims to compare 14 countries with regards to many variables related to healthcare systems. Countries leading in deceased organ donation spend more on health and education, which is associated with increased potential for deceased organ donation. Out-of-pocket expenditure, is also associated with a decrease in deceased organ donation. Countries in SEA are lacking in healthcare resources such as workforce and materials, which are both necessary for a successful transplant program. Most countries in SEA have an excellent foundation for successful organ donation systems, including proper legislation, government support, and brain death laws along with an overall acceptance of brain death diagnosis. Priorities should include improving coordination, donor identification, and healthcare worker education. Countries in SEA have a lot of potential to increase deceased organ donation, especially by investing in healthcare and education. There is no one size fits all for organ donation programs and countries in SEA should focus on their strengths and take cultural differences into consideration when planning interventions.

## Introduction

Around the world, the need for organ transplantation is constantly growing due to an increase in non-communicable diseases and aging populations. Medical advances and expanding health coverage in the past few decades have allowed people to live much longer with their chronic illnesses, but an organ transplant remains the most cost-effective and long-lasting option in many cases [1]. Although organ donation has been steadily increasing in the last couple of decades, there remains great inequalities between different regions around the world. Europe and North America are far ahead of the other regions, with Spain and the US having 49.61 and 36.88 actual deceased organ donors per million population (pmp), respectively in 2019 [2]. In comparison, nations in SEA had some of the lowest rates of deceased organ donors in the world [3], with 3.66 pmp in Thailand and only 0.53 pmp in Malaysia [2]. This gap highlights the importance of establishing a solid framework for organ donation in SEA, which will rely on changes in legislation, education, and healthcare [3]. A lot of research has been done on the reasons why countries in SEA have such low rates of deceased organ donors, but a comparison of healthcare systems between the countries with the highest rates of deceased organ donors and countries in SEA with extremely low rates has never been done. The main purpose of this research is to highlight the similarities and differences between the healthcare systems of countries leading in deceased organ donation and countries in SEA. Furthermore, the authors wanted to identify strengths and weaknesses of each country in order to suggest interventions to increase deceased organ donation.

**FIGURE 1:**
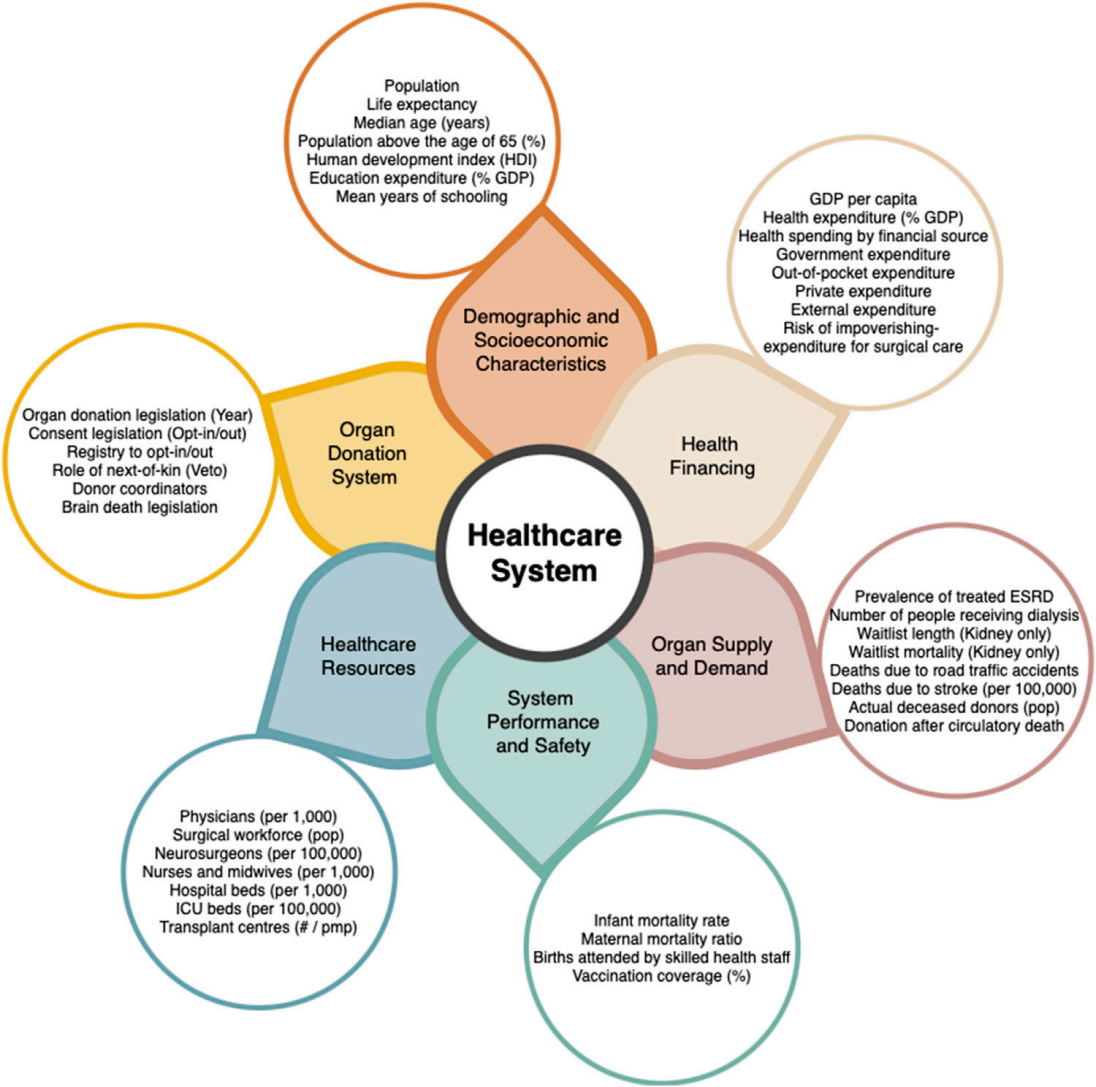
Healthcare system variables possibly related to organ donation.

Healthcare systems worldwide are extremely varied and unique. A combination of resources, population needs, and organizational capacity leads to differences in access and utilization. Variation in deceased organ donation between countries has been proven to be unrelated to medical need [4, 5], but instead correlated with the availability of healthcare resources, a country’s GDP *per capita*, and health expenditure (percentage of GDP spent on healthcare) [4–7]. Intuitively, higher income *per capita* allows for higher health spending and better access to advanced medical technology required for transplantation [5]. Another reason for differences in healthcare system may be due to having different healthcare related priorities due to cultural and social values [8]. Therefore, when comparing countries with different demographics, it is essential to remain aware of the circumstantial differences of each country [8]. A healthcare system is a dynamic and constantly growing mechanism. There are many different aspects that have immense impacts on efficiency and outcomes, and no one healthcare system looks the same. Figure 1 shows the variables chosen to be explored in this research.

The countries chosen for this analysis include the ten countries with the highest rates of deceased donors per million population according to IRODaT 2019, which are Spain, United States, Croatia, Portugal, France, Belgium, Czechia, Finland, Belarus, and Malta [2]. No countries were excluded based on population size or systemic or legislative requirements. The four remaining countries were chosen due to their geographic location (being in SEA) and due to being part of the Organ Donation Initiative Strategies for Southeast Asia (ODISSeA) consortium. ODISSeA’s main objective is to design and implement an academic postgraduate program in organ donation in eight universities across Malaysia, Myanmar, Philippines, and Thailand [3].

## Current Status of Organ Donation in Southeast Asia

SEA continues to experience low rates of deceased organ donors despite seeing a steady increase in economic growth. Inadequate organ donation legislation has led to struggles with organ trafficking and transplant tourism [9], leading to demands towards government officials to make changes regarding healthcare financing, legislation, and medical technology diffusion [10]. The Istanbul declaration of 2008 aimed to decrease illegal practices in organ transplantation, but previous higher rates of donation, which were partially due to transplant tourism, decreased dramatically and have not been able to recover [10]. Below are brief summaries of the status of organ donation in the four countries in SEA studied.

### Malaysia

The healthcare services for a population of 33 million in Malaysia are delivered through public and private providers. Malaysia does not have a national insurance program; however, all citizens get treatments including transplants through centrally funded and administered government health facilities at very low cost [11]. The first organ transplant was performed in 1975 with a living-related kidney transplant and the first deceased kidney transplant was performed the following year [12]. Facilities for kidney, liver, heart, and lung transplants are available in seven public and private hospitals, all located around the capital city. Only public and university hospitals carry out transplants from deceased donors. The National Transplantation Programme is governed by the National Transplantation Council under the Malaysian ministry of health. The National Transplant Resource Centre was established in 1997 to coordinate deceased organ and tissue donation at the national level and is supported by Tissue Organ Procurement teams, which are available in regional hospitals [13]. The practice of deceased donation is legalised by the Human Tissues Act (1974) [14] and supported by the National Fatwa (1970) [15]. Despite efforts to increase organ donation, deceased donation rates remained below 1.0 donor pmp. Living donations make up the majority the organ transplantation [16].

### Thailand

The country of approximately 69.6 million performed its first transplant in 1972 [17]. Thailand now performs kidney, liver, heart and lung transplants in 28 transplant centers across the country [18]. The Organ Donation Center, established in 1994 under the authority of the Thai Red Cross Society, is responsible for overseeing the transplant practice, recovery and distribution of deceased organs, public relations, fundraising, and legal issues [17]. Except for the basic principles set by the Medical Council and the Red Cross, Thailand has no laws specific to organ donation [19]. Three government health coverage schemes, namely, the Civil Servant Medical Beneficiary System, the Social Security Organization, and the Universal Health Coverage Scheme (UCS), cover the entire population. In 2008, the cost of surgery, including post-operative care and immunosuppressive medication, became reimbursable for all citizens following the launch of universal renal replacement therapy program under the UCS [20]. Deceased donation rate improved remarkably from 0.7 in 2005 to 4.8 pmp in 2020 and is now the highest in SEA [2]. The number of kidney transplant from deceased donors exceeds the number of transplants from living donors since 2011 [18]. Unlike Malaysia, both public and private hospitals perform transplant from deceased donors [18]. Organ donation rates have been on the rise thanks to public organ donation campaigns supported by the Thai Royal family; however, shortage of organs still limits the rate of transplantation [18].

### Philippines

The Philippines, with a population of 108.1 million population, recorded only 26 deceased donations between 2017 and 2019 [2]. Philippines has an administratively decentralized public health system, where local governments have full policy and fiscal freedom [21]. The Department of Health (DOH) is the national health agency that develops and regulates national policies and provide tertiary and specialized hospital services [21]. Social health insurance was introduced in 1995 and administered by the Philippine Health Insurance Corporation (PhilHealth) to enhance the nation’s financial risk protection, however it only contributes to a small portion of total health expenses [21]. The Passage of Organ Donation Act of 1991 legalized deceased donation for treatment, research, or medical education by will of the deceased or consent from family members [22]. Philippine Network for Organ Sharing (PhilNOS), which was established in 2010 by the DOH, is the central coordinating body that regulates transplant activities including deceased donation, organ allocation, and maintaining the national registry [9]. Organ Procurement Organizations (OPO) operate under donor service areas designated by PhilNOS responsible for brain death certification, acquiring consent, donor maintenance, retrieval organ and tissues from deceased donors for transplantation [23]. There were 18 accredited transplant centers distributed in different regions of the Philippines [24].

### Myanmar

Myanmar has a shorter history of organ transplantation, having started with kidney transplants in 1995 and liver transplants in 2004 [25, 26]. Currently, transplant for kidney and liver are available in nine hospitals. Myanmar, with a population of 54 million, has universal health coverage through public facilities but national health insurance system is not available [26]. It is an under-resourced country with key challenges in organ transplantation including shortage of immunology transplant laboratories, trained medical personnel, medication, and financial support. Before 2010, there was an average of 4–5 kidney transplants per year. With the help of international experts through joint operations, on-site medical knowledge sharing, and fellowship training programmes, the number increased substantially over the next 10 years. There were 78 kidney transplants performed in 2018, the highest number ever recorded since the launch of the program. Between 2004 and 2021, 56 liver transplants including two from deceased donor were performed [27]. Despite the improvement in transplantation, a deceased donor program has not been established in Myanmar. The Body Organ Donation Law enacted in 2004 and revised in 2015 allows deceased organ donations with the will of the deceased or consent from the relative, but most transplants are nevertheless from living and non-related donors.

## Healthcare System Comparison

### Demographic and Socioeconomic Characteristics

Life expectancy is on average lower in SEA than in countries leading in deceased organ donation, though there are some exceptions, such as Thailand and Malaysia having a higher life expectancy than Belarus. The Human Development Index (HDI) is associated with deceased donation rate, suggesting that a country needs to have a minimum socioeconomic level to set up and support a deceased donor program [9, 10]. Malaysia is classified as having a very high human development along with other countries leading in deceased organ donation. This reflects the country’s high potential to develop efficient deceased donor activities. Thailand and Philippines have high human development, while Myanmar falls under the medium human development category [11]. Finally, countries in SEA have much younger populations compared to countries leading in deceased organ donation; less than 10% of the population in Malaysia, Philippines and Myanmar are aged 65 years and above (See Table 1: Section A).

**TABLE 1:** Healthcare system comparison variables results.

Country	A. Demographic and socioeconomic characteristics								B. Health financing and health spending			C. Health spending by financial source *per capita* in US$ (% total) (2018)			
	Population 2019 (millions)	Life expectancy	Median age	65+ (%)	HDI	Education expenditure (% of GDP)	Mean years of school	Medical schools (pmp)	GDP *per capita* 2019 (USD)	Health expenditure (% GDP) 2019	Risk of impoverishing expenditure for surgical care (% of people at risk)	GGHE-D	OOPS	PVT-D-OOPS	EXT
Spain	47.13	83.49	44.9	19.6	0.90	4.21	10.3	0.91	29,564.7	9.0	0.1	1,926 (70.4%)	606 (22.1%)	204 (7.5%)	0 (0.0%)
United States	328.24	78.79	38.3	16.2	0.93	4.96	13.4	0.59	65,297.5	16.9	0.2	5,356 (50.4%)	1,148 (10.8%)	4,120 (38.8%)	0 (0.0%)
Croatia	4.07	78.42	44.3	20.9	0.85	3.92	11.4	0.98	14,944.4	6.8	0.1	844 (83.2%)	106 (10.5%)	64 (6.3%)	0 (0.0%)
Portugal	10.29	80.68	46.2	22.4	0.86	5.02	9.3	0.78	23,214.0	9.4	0.3	1,361 (61.4%)	654 (29.5%)	198 (8.9%)	2 (0.1%)
France	67.06	82.56	42.3	20.4	0.90	5.45	11.5	0.57	40,496.4	11.3	0	3,441 (73.4%)	434 (9.3%)	815 (17.4%)	0 (0.0%)
Belgium	11.50	81.75	41.9	19.0	0.93	6.41	12.1	0.61	46,345.4	10.3	0	3,723 (75.8)	936 (19.1%)	254 (5.2%)	0 (0.0%)
Czechia	10.67	79.13	43.2	19.8	0.90	3.85	12.7	0.84	23,489.8	7.6	0	1,460 (82.7%)	251 (14.2%)	54 (3.1%)	0 (0.0%)
Finland	5.52	81.79	43.1	22.1	0.94	6.38	12.8	0.91	48,771.4	9.0	0	3,547 (78.6%)	832 (18.4%)	136 (3.0%)	0 (0.0%)
Belarus	9.42	74.23	40.3	15.2	0.82	4.79	12.3	0.42	6,698.0	5.6	0.1	251 (70.5%)	89 (25.0%)	15 (4.2%)	1 (0.3%)
Malta	0.50	82.60	42.6	20.8	0.90	4.82	11.3	4.00	29,737.3	9.0	0	1,748 (63.5%)	944 (34.3%)	61 (2.2%)	0 (0.0%)
Mean	49.44	80.34	42.71	19.64	0.89	4.98	11.71	1.06	32,855.88	9.50	0.08	2,365.7 (71.0%)	600 (19.3%)	592.1 (9.65%)	0.3 (0.04%)
Thailand	69.63	77.15	40.1	12.4	0.78	4.12	7.9	0.33	7,806.7	3.8	4.7	210 (76.1%)	30 (10.9%)	35 (12.7%)	1 (0.4%)
Malaysia	31.95	76.16	30.3	6.9	0.81	4.16	10.4	1.00	11,414.2	3.8	3.5	219 (51.2%)	150 (35.0%)	59 (13.8%)	0 (0.0%)
Philippines	108.12	71.23	25.7	5.3	0.72	2.54	9.4	0.41	3,485.1	4.4	18.6	45 (32.8%)	74 (54.0%)	17 (12.4%)	1 (0.7%)
Myanmar	54.05	67.13	29.0	6.0	0.58	1.93	5.0	0.11	1,407.8	4.8	—	9 (15.3)	45 (76.3%)	0 (0.0%)	5 (8.5%)
Mean	65.94	72.92	31.28	7.65	0.72	3.19	8.18	0.46	6,028.45	4.20	8.93	120.75 (43.8%)	74.75 (44.1%)	27.75 (9.7%)	1.75 (2.4%)

	D. Organ demand and supply								E. System performance and safety				G. Organ donation system					
Country	Prevalence of treated ESRD (pmp)	Dialysis (pmp)	Waitlist active^a^ (pmp)	WaitlistMortality^b^	RTA mortality (pmp)	Stroke mortality (pmp)	Actual deceased donors (ppm)	DCD (pmp)	Infant mortality rate	Maternal mortality ratio	Births attended by skilled health staff	Immunization coverage (%)	Consent	Year	Registry	Next-of-kin can veto decision	In-hospital donor coordinator	Brain death legislation
Spain	1234^c^	587^c^	83.45	—	0.39	0.79	49.61	16.06	2.6	4	—	96.67	Opt-out	1979	No	NA	Yes	Yes
United States	2,354	1,699	184.52	3.88%	1.27	0.58	36.88	8.26	5.6	19	99.1	91.67	Opt-in	1967	Yes	No	Yes	Yes
Croatia	—	610^d^	58.29	2.72%	0.79	1.86	34.63	0	4.1	3	99.9	93.33	Opt-out	1988	Yes	Yes	Yes	Yes
Portugal	2,014	1,265	195.24	0.86%	0.82	1.62	33.8	2.6	3.1	10	98.7	98.67	Opt-out	1993	Yes	—	Yes	Yes
France	1,349	731	128.87	2.05%	0.51	0.67	33.25	6.97	3.8	4	98.1	92.33	Opt-out	1997	Yes	Yes	Yes	Yes
Belgium	1,290^c^	1,481^c^	79.48	2.01%	0.58	0.81	30.3	10.52	2.7	5	99.3	97.00	Opt-out	1986	Yes	No	—	Yes
Czechia	1,128	656	49.02^e^	5.38%^e^	0.59	1.08	27.14	1.79	2.5	1	99.8	95.33	Opt-out	2002	Yes	No	Yes	Yes
Finland	926	367	66.85	0.95%	0.39	1.01	26.23	0	1.9	8	100	93.50	Opt-out	2001	Yes	No	Yes	Yes
Belarus	248^c^	151^c^	19.85	6.32%	0.76	1.80	26.2	0	2.4	1	99.8	97.67	Opt-out	1997	Yes	Yes	—	Yes
Malta	—	600^d^	178.22	5.00%	0.41	0.66	25	0	6.1	0	99.7	97.33	Opt-in	2016	Yes	—	Yes	Yes
Mean	1510.08	943.60	106.76	3.24%	0.65	1.09	32.30	4.62	3.48	5.50	99.38	95.35						
Thailand	2,028	1,885	92.16^f^	—	3.22	0.73	3.66	0	7.7	24	99.1	96.67	Opt-in	None	Yes	Yes	No	Yes
Malaysia	1,412	1,357	161.10^e^	8.92%^e^	2.25	0.62	0.53	0	7.3	23	99.6	97.33	Opt-in	1974	Yes	Yes	No^h^	No
Philippines	224^g^	607^f^	64.74^f^	—	1.20	0.67	0.09	0	21.6	206	84.4	65.67	Opt-in	1992	—	Yes	No	Yes
Myanmar	—	75^f^	—	—	2.04	1.53	0	0	35.8	244	60.2	88.00	n/a	2004	No	NA	No	No
Mean	1221.33	981.06	106.00	—	2.18	0.89	1.07	0.00	18.10	124.25	85.83	86.92						

^a^ Number of people on the waitlist at the end of 2019.

^b^ Number of people who died while on the waitlist over the total number of people who were on the waitlist in 2019.

^c^ Data from 2016 instead of 2018.

^d^ Data from 2019, not 2018.

^e^ Data from 2018 instead of 2019.

^f^ Year of data unknown but published recently.

^g^ Data from 2013.

^h^ Malaysia now has a few hospitals with donor coordinators since 2020. Data in table is based on 2019, to reflect rated of actual deceased organ donors.

Countries in SEA spend less on education and individuals in Thailand and Myanmar receive on average less years of schooling. However, Malaysia does have the greatest number of medical schools pmp after Malta (See Table 1: Section A). Government education expenditure is positively associated with deceased kidney transplant rates and the percentage of the population with higher education significantly associated with higher rates of organ donation [4, 7]. Educational attainment is also significantly associated with willingness to donate [1, 28]. Overall, education is a vital aspect of an efficient organ donation system. Increased spending on education could increase the knowledge about organ donation in the general population and improve the quality of education available to healthcare workers interested in the field of organ donation. The concept of health literacy may also be important, especially since healthcare systems have been becoming more complex and more difficult to navigate [29].

Another vital impact on organ donation are cultural and religious beliefs. In Malaysia, many cite religion to be a reason why they would refuse to become organ donors. However, some of the more common reasons for not wanting to become an organ donor was related to a lack of trust in the healthcare system to use their body in an appropriate manner and a lack of understanding of what organ donation was and why it was such a necessity. Some cultural beliefs such as wanting their body to remain intact after death was also a common response [30]. Strong beliefs surrounding familial involvement in the decision may also be a reason why people do not give consent for donation before death [31].

A study done in Germany comparing organ donation as it relates to Christians, Muslims, Jews, Hindus, and Buddhists showed that most view organ donation as an altruistic and heroic act, as long as certain rules are respected. All except Buddhism had a universal acceptance of the concept of brain death and believed both the donor and family members had the right to decide for the donor. Despite this, many in the study had still not signed a card saying that they accepted to be organ donors. This was largely due to misconceptions or misunderstandings of religious doctrines and a fear of doing something wrong [32].

The countless studies on organ donation, culture, and religion shows the importance of education and campaigns with a highlight on religious acceptance of them. Encouraging individuals to discuss organ donation with friends and family should also be encouraged since familial decision making is so important.

### Health Financing

One of the most important aspects when determining the strength of a healthcare system is undeniably related to money. Countries leading in deceased organ donation have on average 5.5 times higher GDP *per capita* than countries in SEA and spend around 2.25 times more of their GDP on health (health expenditure) (See Table 1: Section B). Countries leading in organ donation spend on average 9.5% of their GDP on health, ranging from 5.6% in Belarus to 16.9% in the United States. Countries from our SEA group spend on average 4.2% of the GDP on health, ranging from 3.8% in Thailand and Malaysia, to 4.8% in Myanmar. We also need to consider the difference in raw GDP, meaning the low percentage is exponentially lower in actual amount of money spent. Increased health expenditure is associated with increased quality of critical care, which is essential for organ donation [33]. Furthermore, individuals living in SEA are much more at risk of impoverishing expenditure due to need of surgical care, a risk that does not exist in countries leading in organ donation.

### Health Spending

**FIGURE 2:**
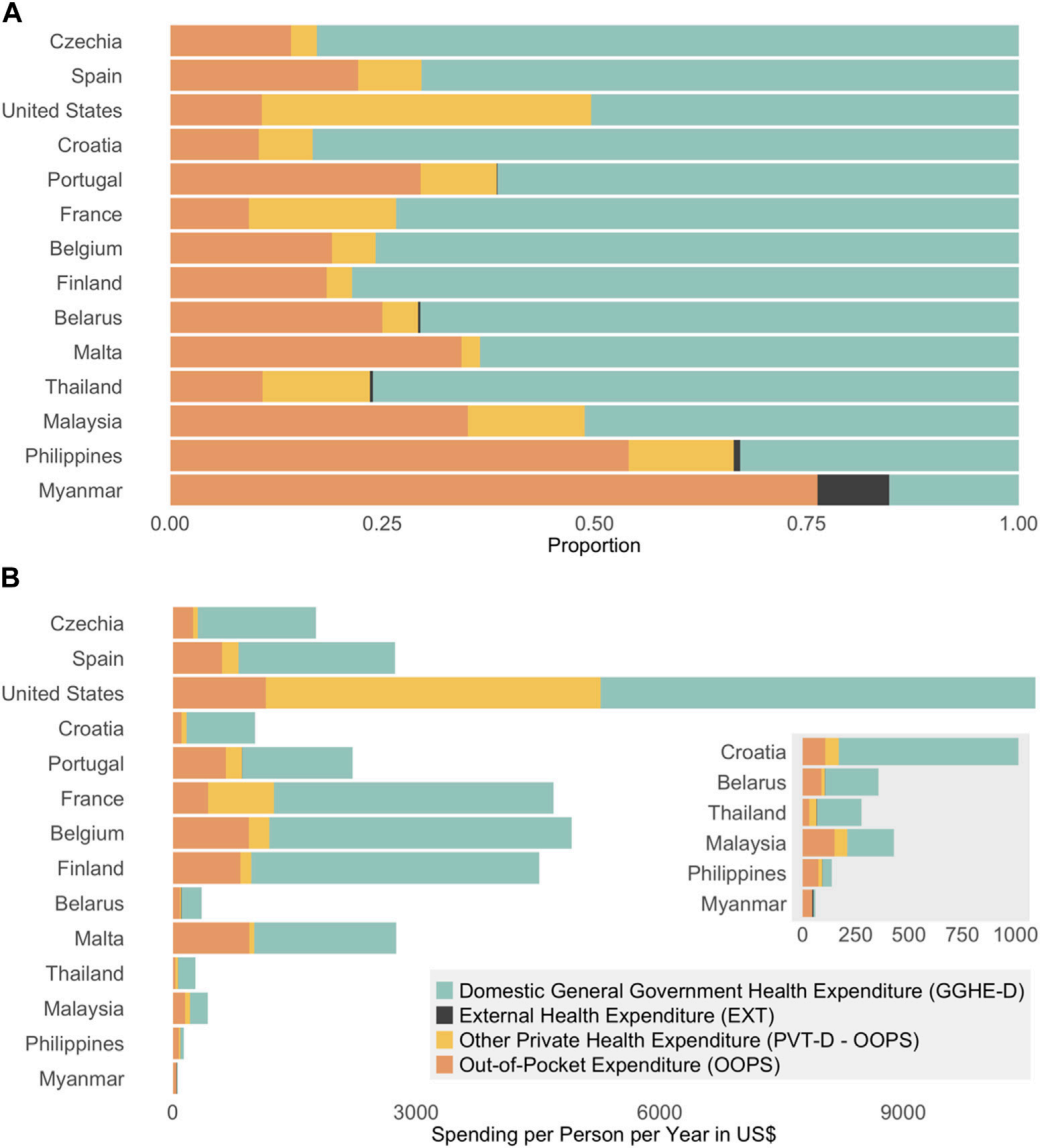
**(A)** proportion of health spending by financial source. **(B)** Health spending by financial source *per capita* in US$.

To better understand health financing, we need to look at the sources of financing, namely, government, external sources, out-of-pocket (household spending), and other private sources such as insurance (See Table 1: Section C; Figure 2A). Government contribution in SEA is fairly low, especially in the Philippines and Myanmar. However, the government in Thailand contributes on average 76%, which is more than any other SEA country and even surpasses some countries leading in organ donation. Percent share of OOPS is much higher in SEA, although the United States has the highest crude OOPS by far, it only accounts for 10.8% of all health financing. This could be due to differences in cost of care in different countries [34]; individuals in the United States pay more for health services, but the government and private sources also contribute more (See Figure 2B). The United States has the highest crude and proportion of spending coming from other private sources due to its notable privatized insurance system. The proportion of financing coming from private sources is much higher in SEA, except Myanmar, which instead has a notable source of funding coming externally.

**FIGURE 3:**
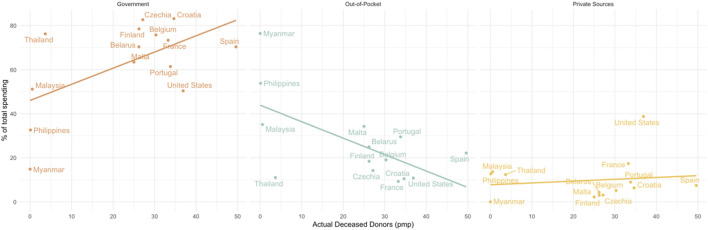
The relationship between actual deceased donors (pmp) and financial source.

Higher government spending (%) and lower OOPS (%) is associated with higher rates of deceased organ transplantation, whereas private health expenditure had no impact on rates of deceased organ transplantation (See Figure 3). By decreasing out-of-pocket costs by either increasing government spending or by increasing access to equitable and efficient private insurance, deceased organ donation capacity may be greatly increased in SEA.

### Organ Demand and Supply

The incidence and prevalence of end-stage-renal disease (ESRD) is increasing globally. This is also leading to an increase in need for dialysis and transplantation. In this 14-country comparison, there is not a big difference in ESRD prevalence between the two groups (See Table 1: Section D). Malaysia and Thailand have higher rates of dialysis than the average for countries leading in organ donation (943.60). Philippines and Myanmar, however, are below that average, possibly due to high out-of-pocket costs for dialysis [34]. Dialysis is a very expensive, long-term treatment, costing generally twice as much as a renal transplant when looking at a time frame of more than 1 year [35]. In countries with government reimbursement for dialysis, such as Thailand and Malaysia, increasing deceased organ donation should be a government goal due to cost-effectiveness.

Waitlist length is difficult to interpret because a low number could represent either a low need for transplantation, an unused waitlist system, or an effective transplant system. Waitlist mortality, represented as the percentage of people who died while waiting for an organ (Waitlist includes total for kidney, liver, heart, lungs, pancreas, and small bowel) out of everyone who was ever on the waitlist in that year, is a better indicator of unmet needs for organ donation. Malaysia has a waitlist mortality of 8.92%, nearly three times larger than the average for countries leading in organ donation. Data for the other three countries in SEA could unfortunately not be found.

Most deceased organ donation occurs after brain death, usually caused by road traffic accident (RTA) injury and stroke [36]. Countries in SEA have on average 3.35 times more deaths from RTA injury (pmp) than countries leading in organ donation but have on average fewer deaths due to stroke (pmp). Donation after circulatory death (DCD) is becoming increasingly common. No country in SEA performs DCD, but 6 of the top 8 countries do as of December 2020, with Croatia and Finland planning to implement legislation in the near future [37]. Finland did have its first DCD transplants in 2021 (IRODaT). Some researchers recommend expanding DCD programs to increase potential donors in countries with currently low rates of deceased organ donation [38, 39]. Unfortunately, instating legislation for DCD is complex and requires a lot of organizational and financial capacity [37]. Furthermore, the need for DCD is mostly due to the decreasing rates of traumatic brain injuries from RTA in developed countries, a problem that SEA is not yet facing [40]. For these reasons, implementing DCD should not be a priority for SEA at this time. However, due to a high number of potential donors due to elevated RTA mortality, donor identification, one of the first steps in the deceased organ donation process, should be prioritized [41]. This comes back to investing in educational programs for healthcare workers.

### System Performance and Safety

Some health indicators are more often used to measure the status of a healthcare system and are widely accepted as representative of a country’s overall health. These often include infant mortality (IMR) and maternal mortality (MMR) [42, 43]. Because most maternal deaths are preventable, they should be close to zero in a safe and effective system [43]. High maternal mortality is often associated with scarcity of health resources and certain political issues such as government corruption [43]. The IMR in Thailand and Malaysia only about twice as high as the average IMR in countries leading in deceased organ donation. However, the IMR is 6 times greater in Philippines and 10 times greater in Myanmar compared to the top 10 countries. MMR follows the same trend, with Thailand and Malaysia being around 4 times greater than the average for countries leading in organ donation, whereas Philippines and Myanmar have a MMR 37.5 times and 44.3 times greater, respectively. Delivery by a skilled birth attendant is a measure of the progress toward eliminating maternal mortality and is commonly used as a measure of access to and safety of healthcare in a country [44]. Almost 100% of births are attended by a skilled healthcare professional in Thailand and Malaysia, like all countries leading in organ donation, whereas only 84.4% of births in Philippines and 60.2% of births in Myanmar are attended by a skilled healthcare professional. Average infant immunization rates (Hepatitis B, Measles, and DTP) are also as high in Thailand and Malaysia, but Myanmar and Philippines are still lacking in this area (See Table 1: Section E). The system performance between countries is very different in SEA, namely, Malaysia and Thailand appear to be far ahead of Myanmar and Philippines. Malaysia and Thailand have a lot of potential to increase deceased organ donation through slight alterations in legislation and education, whereas Myanmar and Philippines may need a few more years to catch up and organ donation may not be a priority at this time. Major issues of safety and access first need to be addressed.

### Healthcare Resources

**FIGURE 4:**
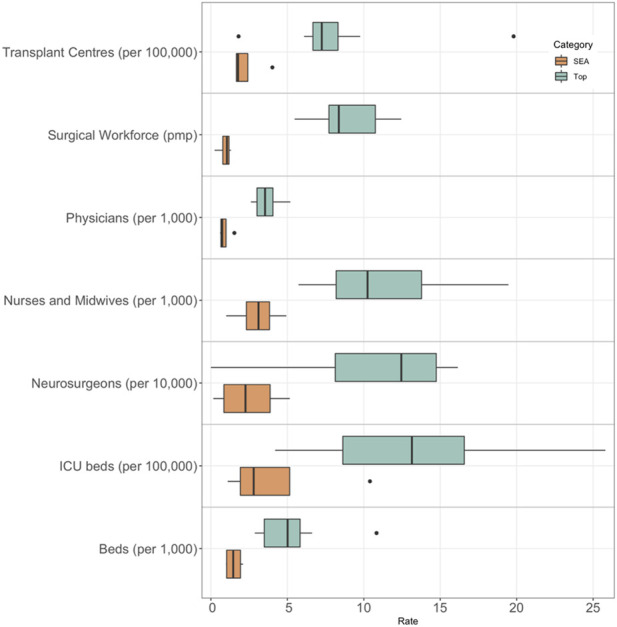
Healthcare resources in SEA compared to countries leading in deceased organ donation.

Some of the biggest barriers for obtaining organ donors include poor hospital infrastructure, missing manpower, and inability to identify and support brain dead donors [45]. On average, countries leading in organ donation have 4.1 times more physicians, 9.8 time more surgical workforce, 4.6 times more neurosurgeons, and 3.6 times more nurses and midwives than countries in SEA. Regarding materials, countries leading in organ donation have on average around 3.5 times more beds, ICU beds, and transplant centres (pmp). Data for healthcare resources can be found in Supplementary Table S2 and are visually presented in Figure 4.

The availability of staff and materials has a very negative effect on the organ donation process. The “death to donation to transplantation process” suggested by Manzano in 2014 relies heavily on availability of healthcare professionals for donor identification and retrieval, consent to donation, and organ retrieval [41]. The lack of nurses and doctors in SEA severely decreases the ability of staff to fulfill organ donation related tasks on top of their regular tasks. To optimize the process, countries in SEA should focus on incentivising people to enter healthcare professions. Another option is to use non-medical professionals to carry out donor coordinator tasks, like what is done in the United States. Although donor coordinators should ideally be given enough time to carry out donor coordinator related task, a minimum requirement would be to pay them for the work they do, either per patient or per hour. This is done in most countries leading in organ donation who do not have donor coordinator only positions.

The organ donation process is also dependent on expensive materials for donor assessment, donor maintenance, and organ storage and transportation [41]. A lack of essential equipment such as hospital beds and ICU beds could be detrimental to deceased organ donation [38]. If there are insufficient beds, the hospital cannot justify keeping a bed for even just several hours to wait for a recipient of the organs. However, the use of ICU beds in the organ donation process varies greatly from country to country, meaning some countries may have a more efficient way of managing ICUs and distributing patients across different levels of care units [46].

This can be seen with the leader of deceased organ donation, Spain, having one of the lowest number of ICU beds per 100,000 population in the top 10 leading countries, having even fewer ICU beds per 100,000 population than Thailand (See Supplementary Table S2). This demonstrates that although a baseline ICU capacity is needed, efficient management of assessing and treating potential donors is just as important if not more. This is due to other necessary components of an efficient transplant system such as institutional reformation, quality assurance, reimbursement schemes and comprehensive training programs [47]. The organizational components of Spain’s transplant system, such as donor coordinators, may also contribute to the efficiency of their ICUs without the need for as many beds as other countries leading in organ donation. Another non-medical but closely related variable that organ donation is highly dependent on is access to efficient transport. In Spain, individuals in rural areas needing transplant can be transported by helicopter, whereas this type of rapid transport is not available in SEA. This rapid transportation system makes for an extremely efficient transplant network.

### Organ Donation System

Every country has a unique combination of laws and regulations regarding practices, coordination, and consent (See Table 1: Section G). All countries in SEA have opt-in consent systems, except Myanmar, which lacks regulations to be considered either. Countries leading in deceased organ donation are mostly opt-out countries, except US and Malta. A lot of research has been done comparing opt-in versus opt-out countries and found that although deceased donor rates are higher in opt-out countries, the difference is not significant and is most likely not solely due to the consent legislation, but rather due to other organizational components [7, 48, 49]. There does not seem to be an association between rates of organ donation and the year of initial donation legislation, since Malaysia was one of the first to implement legislation, even before Spain. However, organ donation did not take off in Spain until the creation of the National organization of transplantation (ONT) in 1989 [50]. This suggests that merely having a legislation or law regarding organ donation is not sufficient to increase organ donation and having organizational components are mandatory for efficiency and success.

The usefulness of registries is also a topic of debate. Most countries have a registry, either to opt-in or opt-out, or in the case of Belgium, both opt-in and opt-out. Donor registries can be useful not only for identifying potential donors, but also to promote public awareness [51]. However, since Spain does not have a registry, we can confidently say that the success of an organ donation system does not depend on the presence of a registry, though this may be truer for opt-out systems. There has never been research done on the effectiveness of a registry and how many donors come from checking the registry compared to asking family for consent. Obtaining consent from family members is considered one of the essential elements of a successful organ donation system [51]. In most countries, the final decision is ultimately up to the next-of-kin, also known as soft opt-out [52]. In Belgium, however, an individual’s name on either the opt-in or opt-out registry is legally binding. So even if the family knows their loved one had changed their mind, the organs cannot be retrieved. In Malaysia and Thailand, consent to donate is always asked from the next-of-kin whether the individuals’ name is on the registry or not. With this, individuals who have opted-in can still become non-viable donors due to declined family consent. Some believe this “overrule” could jeopardize the trust in the donation system, since individuals will not feel like their wishes will be respected [1]. Many countries with hard opt-out legislation still use a soft opt-out approach because not following the wishes of the family leads to more negative publicity that could put organ donation in a negative light.

Another vital component of the organ donation system are donor coordinators. Spain is often cited as the poster-child of deceased organ donation, having the most successful program in the world [2]. The “Spanish Model” relies on access to higher education to support doctors and nurses working in ICUs who have high exposure to potential donors [40]. With advanced education in donor identification, brain death diagnosis, donor management, family approach, grief counselling, refusal management, and organ allocation, healthcare professionals are more familiar and have a more positive view of the organ donation process [53, 54]. In Spain, donor coordinators are often physicians familiar with the critical care unit and are highly motivated about organ donation. This maximizes efficiency since they may already have a relationship with the families, they approach to request donation consent [55]. Donor coordinators are different from transplant coordinators, who often work on dialysis units and support recipients of organs. Many countries have followed Spain’s example and have implemented in-hospital donor coordinators such as Croatia [56], leading to a dramatic increase in deceased organ donation. However, Germany also attempted to implement this type of in-hospital coordinator in 2012 but did not see the same success [40]. The ODISSeA project allowed a group of physicians from SEA to attend seminars in Spain in 2019 to help develop a post-graduate organ donation program in SEA. Some trained healthcare professionals in organ donation started working in hospitals as acting donor coordinators at the start of 2020 and, despite the negative impacts of COVID-19 on the healthcare system, Malaysia saw an increase from 0.53 pmp in 2019 to 0.9 pmp in 2020. Many hope that by increasing the availability of these programs in universities across SEA and implementing more in-hospital donor coordinators, countries could continue to see an increase in deceased donor transplantation.

Increasing organ donation relies heavily on both professional and public acceptance of brain death [46]. The lack of awareness around this concept can lead to a significant reduction in potential donors as well as a decrease in donor identification [45]. Although most countries have some laws regarding brain death diagnosis, these vary slightly between different countries [57]. Brain death legislation was introduced a lot later in most Asian countries, where cultural resistance and fear of abuse remain serious issues [39]. Brain death is legally recognized in Thailand (1989), Malaysia (2006), Philippines (1991) and Myanmar (2009), but there is no official law in Malaysia and Myanmar [58]. Brain death diagnosis requires multiple exams separated by a determined time and the presence of 2–3 doctors with varying qualifications (neurologist/neurosurgeon, anesthesiologist, intensivist, internist). These criteria are the same in countries leading in organ donation, but the availability of such specialists is a lot lower in SEA. Brain death is becoming more accepted among both health professionals and the general population in SEA. Nevertheless, religion and culture are still some of the main reasons for family objection to donation [59].

## Discussion

The countries in this comparison come from a variety of economic and developmental backgrounds. This makes comparison very difficult. For example, even in SEA, Thailand and Malaysia are very different from Philippines and Myanmar regarding financial and resource capacity. In the group of countries leading in deceased organ donation, countries are more homogeneous, with Belarus being a unique example. Belarus is the only upper-middle income country in the group of top ten countries in deceased organ donation. This is possible evidence that Thailand and Malaysia, which are both also upper-middle income countries, have the capacity to increase deceased organ donation through organizational changes. Due to cultural, social, and economic differences between the four SEA countries, every country has strengths and weaknesses regarding deceased organ donation capacity and should implement strategies to increase donation based on those particularities (See Table 2).

**TABLE 2:** SWOT analysis of increasing deceased organ donation in 4 SEA countries.

	Strengths	Weaknesses	Opportunities	Threats
Thailand	- Highest actual deceased donors pmp in SEA - High HDI - Second fastest growing GDP and GDP *per capita* in SEA - High government spending (%) on health - Low out-of-pocket spending - Highest rate of RTA mortality = high potential for brain dead donors - Highest rate of surgical workforce, beds, neurosurgeons, and ICU beds in SEA - Highest rate of transplant centres in SEA - Decreasing IMR and MMR - High access and safety of healthcare	- Low level of population education - High prevalence of ESRD and dialysis = high need for transplantation - Low levels of doctors and nurses	- Focus on organ donation for cost-effectiveness, since so many people require dialysis - To address low levels of doctors and nurses, either encourage more to enter healthcare professions or use non-medical staff as donor coordinators - Infrastructure (transplant centres) is already pretty good, so just focus on organizational components to increase donor identification and referral: consider Spanish model donor coordinators	
Malaysia	- Very high HDI - High GDP *per capita* - High government spending (%) on education - Highly educated population (mean years of school) - Highest number of medical schools pmp - Highest rate of physicians in SEA - Good monitoring system for disease, treatment, and organ donation activity - Decreasing IMR and MMR - High access and safety of healthcare	- Excessive out-of-pocket costs - High prevalence of ESRD and dialysis = high need for transplantation - High waitlist mortality	- Continue training physicians to be donor coordinators by making more programs available throughout the country - Focus on population education through educational campaigns to raise awareness about organ donation - Focus on organ donation for cost-effectiveness, since so many people require dialysis - Reduce out-of-pocket spending by either increasing government spending or increasing access to private insurance	- Population level superstitions related to organ donation [28] - Slowest growing GDP in SEA
Philippines	- High HDI - Fastest growing GDP (80% 10 year increase) and GDP *per capita* (57% 10 year increase) in SEA - Highest ratio of nurses to population in SEA - Good education despite low GDP *per capita* and low education expenditure	- Lowest level of physicians and hospital beds - Inadequate diseases, treatment, and organ donation activity surveillance - High out-of-pocket spending	- Use nurses as donor coordinator to compensate for the low levels of physicians - Increase surveillance of supply and demand of transplantation along with illness to better track progress	- Issues with organ trafficking and transplant tourism [10]
Myanmar	- Relatively fast-growing GDP *per capita* - Medical professionals remain motivated and hopeful, participating in ODISSeA and other research contributing to finding ways to increase organ donation in the country - Lowest rates of actual deceased donors per population means the greatest potential to increase	- Low HDI - Low GDP *per capita* - Low education attainment - Low government health spending (15%) - High out-of-pocket spending (76%) - No private sources of health financing - Inadequate diseases, treatment, and organ donation activity surveillance	- Focus on education initiative for both the general population and healthcare professionals	- Political instability [56] - Health-seeking behaviour rooted in traditional health beliefs [56]

Thailand currently has the highest number of deceased donors pmp in SEA. They have a high HDI and the second fastest growing GDP and GDP *per capita* in SEA after the Philippines. They already have high government spending on health and therefore low out-of-pocket costs for health. Along with the highest rates of surgical workforce, hospital beds, neurosurgeons, and ICU beds in SEA, they also have the highest rates of transplant centres in SEA. With a decrease in IMR and MMR and an increase in access and safety of healthcare, Thailand is on its way to catching up to other countries leading in organ donation. Some things standing in the way of Thailand perfecting its transplant program include lower than average levels of population education, low levels of doctors and nurses, and a high prevalence of ESRD and dialysis, meaning an elevated need for organ donation. The Thai government should focus on organ donation based on cost-effectiveness; encouraging people to become organ donors after death to help the thousands of people on dialysis. They also need to address the low levels of doctors and nurses, encouraging people to enter the profession. Luckily, Thailand already has an incredible infrastructure and just needs to fine tune its organizational components to increase donor identification and referral. We recommend funding University level programs for the training of donor coordinators that could increase the efficiency of Thailand’s transplant program.

Malaysia also has a lot of potential, considering its very high HDI, high GDP *per capita*, and high spending on education leading to a highly educated population and the most number of medical schools pmp. This in turn leads to Malaysia having the highest rates of physicians. Malaysia is also catching up the high-income countries leading in organ donation with its good monitoring system for disease, treatment, and organ donation activity, decreasing IMR and MMR, and increase in access and safety of healthcare. Weaknesses include high out-of-pocket costs for healthcare, a high prevalence of ESRD and dialysis, and a high waitlist mortality. Malaysia should prioritize developing an efficient organ donation system due to so many people requiring dialysis. They should focus on training physicians to be donor coordinators by making more programs available throughout the country. The government should also focus on population education through educational campaigns to raise awareness about organ donation. Finally, the Malaysian government should focus on reducing out-of-pocket spending by either increasing government spending or increasing access to private insurance.

The Philippines has a high HDI with the fastest growing GDP and GDP *per capita* in SEA. They also have the highest ratio of nurses in SEA and high levels of population education despite having a low GDP *per capita* and low education expenditure. What weakens the healthcare system is a lack of physician and hospital beds, high out-of-pocket spending for healthcare, and inadequate diseases, treatment, and organ donation activity surveillance. We recommend the Philippines to nevertheless focus on training donor coordinators but also include nurses at potential donor coordinators to compensate for the low levels of physicians. Increasing surveillance will also help in the efficiency of the transplant system. As a final comment, the Philippines has struggled with organ trafficking and transplant tourism, especially in the past, creating a threat to creating an efficient organ donation program [10]. New legislation has made it more difficult to illegally sell organs, but the population still has some negative views towards the practice in general.

Myanmar may have the lowest rates of actual deceased donors pmp but medical professionals in the country remain motivated and hopeful, participating in ODISSeA and other research contributing to finding ways to increase organ donation in the country. Unfortunately, they do have the lowest rates on almost all indicators presented in this review and have a long way to go to catch up to the other 3 SEA countries in this review but by focusing primarily on education, both of medical professionals and the general population, they can develop their transplant program with the help of countless motivated healthcare professionals. Some threats to developing an efficient organ donation program include political instability [60] and health-seeking behaviour rooted in traditional health beliefs [60].

### Limitations of the Review

This research is a very broad overview of healthcare system variables in relation to organ donation capacity. The limited number of countries makes it difficult to make conclusions regarding concrete areas in need of improvement, but hopefully the research highlights many areas of interest for future research. Another major limitation is the lack of some data, especially for the Philippines and Myanmar. These countries often do not report some disease, treatment, and organ donation data due to lack of advanced surveillance systems. Furthermore, we could not get an interview with a representative from each country and for the countries we did get further input, it was from one single expert. Finally, using globally reported variables is also problematic due to not being able to control for variation in data collection. This is especially problematic when taking variables from different sources, such as was done for ICU beds and prevalence of ESRD and dialysis.

## Conclusion

Organ transplantation is a lifesaving practice that increases the quality of life of those lucky enough to receive one. Deceased organ donation is a very efficient way of mitigating organ waitlists. Although some countries have been able to increase efficiency and maximize their potential by using their strengths, other countries have fallen behind. Countries in SEA have a lot of unused potential which could be utilized by having government support through financial inputs in healthcare. Organ donation education for healthcare workers, such as the initiation of the ODISSeA (Organ Donation Innovative Strategies in Southeast Asia) [3] in Malaysia, Philippines, Myanmar, and Thailand, is an essential part of any developing nation regardless of their resources and limitation.

Due to cultural and economic differences, countries in SEA have different strengths and weaknesses, and should focus on these when planning interventions. There is no one-size-fits-all for organ donation systems; the priority is to find the system that works the best with what each country has to offer.

## Abbreviations

DCD, donation after circulatory death; EXT, external health expenditure; GDP, gross domestic product; GGHE-D, domestic general government health expenditure; ICU, intensive care unit; IMR, infant mortality rate; MMR, maternal mortality ratio; OOPS, out-of-pocket spending; pmp, per million population; PVT, private health expenditure; RTA, road traffic accident; SEA, Southeast Asia.
