# Supplementary material for: Healthcare System Impact on Deceased Organ Donation and Transplantation: A Comparison Between the Top 10 Organ Donor Countries With 4 Countries in Southeast Asia
Source: Transpl Int. 2023 Aug 30;36:11233. doi: 10.3389/ti.2023.11233 (PMC10498995; doi:10.3389/ti.2023.11233)
Supplement: Supplementary file 1 [file Table1.docx]

| **Supplemental Table 1 : Information Source** | | | |  |  |
| --- | --- | --- | --- | --- | --- |
| **Section** | **Indicator** | **Year** | **Definition** | **Source** | **Available at:** |
| **Demographics and Socioeconomic Characteristics** | UN GNI per Capita | 2018 | Countries are classified by their level of development as measured by per capita gross national income (GNI)   - Low-income = GNI < $1,025 - Lower-middle income = GNI between $1,026 and $3,995 - upper-middle-income = GNI between $3,996 and $12,375 - High-income = > $12,375   GNI per capita in dollar terms is estimated using the World Bank Atlas method | The annex was prepared by the Economic Analysis and Policy Division (EAPD) of the Department of Economic and Social Affairs of the United Nations Secretariat (UN DESA). It is based on information obtained from the Statistics Division and the Population Di- vision of UN DESA, as well as from the five United Nations regional commissions, the United Nations Conference on Trade and Development (UNCTAD), the International Monetary Fund (IMF), the World Bank, the Organization for Economic Cooperation and Development (OECD), Eurostat and national sources. | [United Nations](https://www.un.org/development/desa/dpad/wp-content/uploads/sites/45/WESP2020_Annex.pdf) |
|  | UN Country classification |  | The WESP classifies all countries of the world into one of three broad categories: developed economies, economies in transition and developing economies. The composition of these analytical groupings, specified in tables A, B and C, is intended to reflect basic economic country conditions, and are not strictly aligned with the regional classifications defined by the Statistics Division of UN DESA known as M49. |  | [United Nations](https://www.un.org/development/desa/dpad/wp-content/uploads/sites/45/WESP2020_Annex.pdf) |
|  | Life Expectancy | 2019 | Life expectancy at birth indicates the number of years a newborn infant would live if prevailing patterns of mortality at the time of its birth were to stay the same throughout its life. | (1) United Nations Population Division. World Population Prospects: 2019 Revision, or derived from male and female life expectancy at birth from sources such as: (2) Census reports and other statistical publications from national statistical offices, (3) Eurostat: Demographic Statistics, (4) United Nations Statistical Division. Population and Vital Statistics Report (various years), (5) U.S. Census Bureau: International Database, and (6) Secretariat of the Pacific Community: Statistics and Demography Programme. | [World Bank](https://data.worldbank.org/indicator/SP.DYN.LE00.IN) |
|  | Median age | 2020 | Age that divides the population in two parts of equal size, that is, there are as many persons with ages above the median as there are with ages below the median. It is expressed as years. | United Nations | [United Nations](https://population.un.org/wpp/Download/Standard/Population/) |
|  | Population 65+ | 2019 | Population ages 65 and above as a percentage of the total population. Population is based on the de facto definition of population, which counts all residents regardless of legal status or citizenship. | World Bank staff estimates using the World Bank's total population and age/sex distributions of the United Nations Population Division's World Population Prospects: 2019 Revision. | [World Bank](https://data.worldbank.org/indicator/SP.POP.65UP.TO.ZS?view=chart) |
|  | Human Development Index (HDI) | 2019 | A composite index measuring average achievement in three basic dimensions of human development—a long and healthy life, knowledge and a decent standard of living (Scale 0-1, 1=Best). | United Nations | [United Nations](http://hdr.undp.org/en/content/download-data) |
|  | Government Expenditure on Education (% of GDP) | 2017  Except  2019: Myanmar  Malaysia  USA  2013: Thailand  2009: Philippines | General government expenditure on education (current, capital, and transfers) is expressed as a percentage of GDP. It includes expenditure funded by transfers from international sources to government. General government usually refers to local, regional and central governments. | UNESCO Institute for Statistics ( [uis.unesco.org](http://uis.unesco.org/) ) | [World Bank](https://data.worldbank.org/indicator/SE.XPD.TOTL.GD.ZS?view=chart) |
|  | Mean years of schooling | 2020 | Average number of years of education received by people ages 25 and older, converted from education attainment levels using official durations of each level. | United Nations | [United Nations](http://hdr.undp.org/en/content/download-data) |
| **Health Financing and Health Spending** | GDP per capita (Current US$) | 2019 | GDP per capita is gross domestic product divided by midyear population. GDP is the sum of gross value added by all resident producers in the economy plus any product taxes and minus any subsidies not included in the value of the products. It is calculated without making deductions for depreciation of fabricated assets or for depletion and degradation of natural resources. Data are in current U.S. dollars. | World Bank national accounts data, and OECD National Accounts data files. | [World Bank](https://data.worldbank.org/indicator/NY.GDP.PCAP.CD?view=chart) |
|  | Health Expenditure (% GDP spent on health) | 2018 | Level of current health expenditure expressed as a percentage of GDP. Estimates of current health expenditures include healthcare goods and services consumed during each year. This indicator does not include capital health expenditures such as buildings, machinery, IT and stocks of vaccines for emergency or outbreaks. | World Health Organization Global Health Expenditure database ( [apps.who.int/nha/database](http://apps.who.int/nha/database) ). | [World Bank](https://data.worldbank.org/indicator/SH.XPD.CHEX.GD.ZS?end=2018&start=2018&view=map&year=2018) |
|  | Health spending by financial source | 2018 | Health care is financed through a mix of financing arrangements including government spending and compulsory health insurance (“Government/compulsory”) as well as voluntary health insurance and private funds such as households’ out-of-pocket payments, NGOs and private corporations (“Voluntary”). This indicator is presented as a total and by type of financing (“Government/compulsory”, “Voluntary”, “Out-of-pocket”) and is measured as a share of GDP, as **a share of total health spending** and in USD per capita (using economy-wide PPPs) | WHO | [WHO: Global Health Expenditure Database](https://apps.who.int/nha/database/Select/Indicators/en) |
|  | Risk of impoverishing expenditure for surgical care (% of people at risk) | 2019 | The proportion of population at risk of impoverishing expenditure when surgical care is required. Impoverishing expenditure is defined as direct out of pocket payments for surgical and anaesthesia care which drive people below a poverty threshold (using a threshold of $1.90 PPP/day). | The Program in Global Surgery and Social Change ( PGSSC ) at Harvard Medical School ( [pgssc.org](http://pgssc.org/) ) | [World Bank](https://data.worldbank.org/indicator/SH.SGR.IRSK.ZS) |
| Burden of Kidney Disease, Need for Transplantation and Donor Availability | Prevalence of Treated ESRD (pmp) | 2018: Sll other  2016: Spain  Belgium  Belarus  2013: Philippines | Prevalence of treated ESRF are the total number of people in that country currently being treated for ESRD  Missing: Croatia, Malta, and Myanmar | USRDS | [USRDS 2018](https://adr.usrds.org/2020/end-stage-renal-disease/11-international-comparisons)  [USRDS 2016](https://www.usrds.org/media/1738/v2_c11_intcomp_18_usrds.pdf)  [USRDS 2013](https://www.usrds.org/media/1564/vol2_13_international_15.pdf) |
|  | Dialysis (pmp) | 2019: Croatia  Malta  2018: All Other  2016: Spain  Belgium  Belarus  Other: Philippines  Myanmar | Number of people currently receiving dialysis (pmp) | USRDS & Others | [USRDS 2018](https://adr.usrds.org/2020/end-stage-renal-disease/11-international-comparisons)  [USRDS 2016](https://www.usrds.org/media/1738/v2_c11_intcomp_18_usrds.pdf)  [USRDS 2013](https://www.usrds.org/media/1564/vol2_13_international_15.pdf)  Philippines: [Tang (2019)](https://www.ajkd.org/action/showPdf?pii=S0272-6386%2819%2930940-0)  Myanmar: [Naganuma (2012)](https://www.mdpi.com/2673-8236/1/1/9)  Malta and Croatia:  [Newsletter Transplant: International Figures on donation and transplantation 2019/20](https://freepub.edqm.eu/publications) |
|  | Waitlist | 2019  2018: Malaysia  Czechia Republic | Number of people waiting for an organ transplant (per 100,000) | Council of Europe | [Newsletter Transplant: International Figures on donation and transplantation 2019/20](https://freepub.edqm.eu/publications) |
|  | Waitlist Mortality | 2019  2018: Malaysia  Czechia | Number of people who were on the waitlist but died during the year divided by the total number of people ever on the waitlist during that year (including people who were removed, died, or got a transplant) | Council of Europe | [Newsletter Transplant: International Figures on donation and transplantation 2019/20](https://freepub.edqm.eu/publications) |
|  | RTA mortality (pmp) | 2019 | Mortality caused by road traffic injury is estimated road traffic fatal injury deaths per 100,000 population -> data transformed to be pmp | World Health Organization, Global Health Observatory Data Repository ( [apps.who.int/ghodata](http://apps.who.int/ghodata/) ). | [World Bank](https://data.worldbank.org/indicator/SH.STA.TRAF.P5?view=chart) |
|  | Mortality caused by stroke (pmp) | 2019 | Mortality caused by stoke Including ischemic stroke, intracerebral hemorrhage, and subarachnoid hemorrhage -> data transformed to be pmp | GBD | [GHDx](http://ghdx.healthdata.org/gbd-results-tool) |
|  | Actual Deceased Donors | 2019 | Actual deceased donors per million | IRODaT | [IRODaT](https://www.irodat.org/?p=database#data) |
|  | DCD (pmp) | 2019 | Deceased donors after cardiac death per million population | IRODaT | [IRODaT](https://www.irodat.org/?p=database#data) |
| **System Performance and Safety** | Infant Mortality Rate | 2019 | Infant mortality rate is the number of infants dying before reaching one year of age, per 1,000 live births in a given year. | Estimates developed by the UN Inter-agency Group for Child Mortality Estimation ( UNICEF, WHO, World Bank, UN DESA Population Division ) at [childmortality.org](http://childmortality.org/). | [World Bank](https://data.worldbank.org/indicator/SP.DYN.IMRT.IN) |
|  | Maternal Mortality Ratio | 2017: Finland  Portugal  2016: All other  2015: Belgium  France  Malta  2014: Belarus  Malaysia  2012: Philippines | Maternal mortality ratio is the number of women who die from pregnancy-related causes while pregnant or within 42 days of pregnancy termination per 100,000 live births. | The country data compiled, adjusted and used in the estimation model by the Maternal Mortality Estimation Inter-Agency Group ( MMEIG ). The country data were compiled from the following sources: civil registration and vital statistics; specialized studies on maternal mortality; population based surveys and censuses; other available data sources including data from surveillance sites. | [World Bank](https://data.worldbank.org/indicator/SH.STA.MMRT.NE) |
|  | Births attended by skilled health staff | 2018: Finland  France  Portugal  USA  2017: Czech Republic  Croatia  Malta  Malaysia  Philippines  2016: Myanmar  Thailand  2014: Belarus  1999: Belgium  Unavailable = Spain | Births attended by skilled health staff are the percentage of deliveries attended by personnel trained to give the necessary supervision, care, and advice to women during pregnancy, labor, and the postpartum period; to conduct deliveries on their own; and to care for newborns. | UNICEF, State of the World's Children, Childinfo, and Demographic and Health Surveys. | [World Bank](mailto:https://data.worldbank.org/indicator/SH.STA.BRTC.ZS) |
|  | Immunization coverage | 2019 | Percent of 1 year-olds who are vaccinated against Hepatitis B, Measles, and DPT (Average of the three) | WHO and UNICEF ( [who.int/immunization/monitoring_surveillance/en](http://who.int/immunization/monitoring_surveillance/en) ). | [World Bank](https://data.worldbank.org/indicator/SH.IMM.IDPT) |
|  |  |  |  |  |  |
| **Healthcare Resources** | Physicians per 1,000 | 2018: Czech Republic  France  Myanmar  Thailand  2017: Belgium  Spain  Philippines  Portugal  USA  2016: Finland, Croatia  2015: Malta, Belarus, Malaysia | Physicians include generalist and specialist medical practitioners. | World Health Organization's Global Health Workforce Statistics, OECD, supplemented by country data. | [World Bank](https://data.worldbank.org/indicator/SH.MED.PHYS.ZS) |
|  | Specialist surgical workforce (per 100,000) | 2017: Malta  2016: Malaysia  2015: Belgium  France  Croatia  Portugal  USA  2014: Belarus  Spain  Finland  Philippines  Thailand  2013: Czech Republic  Myanmar | Specialist surgical workforce is the number of specialist surgical, anaesthetic, and obstetric (SAO) providers who are working in each country per 100,000 population. | Data collected by the Lancet Commission on Global Surgery ( lancetglobalsurgery.org ); Data collected by WHO Collaborating Centre for Surgery and Public Health at Lund University from various sources including Ministries of Health or equivalent national regulatory bodies, national official entities such as medical councils, Eurostat, OECD, WHO Euro Health For All Database, WHO EURO Technical resources for health Database; BMJ Glob Health. | [World Bank](https://data.worldbank.org/indicator/SH.MED.SAOP.P5) |
|  | Neurosurgical workforce (per 100,000) | 2016 | Density of neurosurgeons per 100,000 population  Data for Malta was not available at the original source, but a quick search for neurosurgeons in Malta gave at least 3 neurosurgeons.  <https://deputyprimeminister.gov.mt/en/MDH/Pages/MDH-Neuro-Surgical-Ward.aspx>  <https://stjameshospital.com/doctors-timetable/> | World Federation of Neurosurgical Societies | [World federation of neurological societies](https://www.wfns.org/menu/61/global-neurosurgical-workforce-map?fbclid=IwAR1-izEH7sDJDbgBDAtRV7O2Y8ETi1cv2N9fzUS0XaLdovAA8mhvi8NWWmQ) |
|  | Nurses and Midwives (per 1,000) | 2018: Belgium  France  Malta  Myanmar  Philippines  Thailand  2017: Czechia  Spain  Malaysia  Portugal  USA  2016: Finland, Croatia  2015: Belarus | Nurses and midwives include professional nurses, professional midwives, auxiliary nurses, auxiliary midwives, enrolled nurses, enrolled midwives and other associated personnel, such as dental nurses and primary care nurses | World Health Organization's Global Health Workforce Statistics, OECD, supplemented by country data. | [World Bank](https://data.worldbank.org/indicator/SH.MED.NUMW.P3) |
|  | Hospital beds (per 1,000) | 2019: Belgium  2018: Czechia  Spain  Finland  France  Philippines  2017: Croatia  Malta  Myanmar  Malaysia  USA  2014: Philippines, Belarus  2010: Thailand | Hospital beds include inpatient beds available in public, private, general, and specialized hospitals and rehabilitation centers. In most cases beds for both acute and chronic care are included. | Data are from the World Health Organization, supplemented by country data. | [World Bank](https://data.worldbank.org/indicator/SH.MED.BEDS.ZS) |
|  | ICU beds (per 100,000) | Phua: 2017  Rhodes: 2010-2011  OECD: 2020 | Number of ICU beds per 100,000 population | Journal Article(s) | Phua (2020)  Rhodes (2012)  OECD |
|  | Transplant centers | 2019: All other  2018: Malaysia  Czechia  Other: Thailand  Philippines | Number of hospitals which act as transplant centers - perform transplants (By country and organ - per 100,000) | Council of Europe | [Newsletter Transplant: International Figures on donation and transplantation 2019/20](https://freepub.edqm.eu/publications) |
| **Organ Donation** | Consent Legislation |  | Opt-in/Opt-out | Journal Article(s) | Cotrau  Shepherd  Martphol |
|  | Year of Legislation | 2012 | The year that the legislation was put in place as well as the dates that it was revised (in parentheses) | Journal Article(s) | [Rosenblum (2012)](https://www.ncbi.nlm.nih.gov/pmc/articles/PMC3363979/) |
|  | Registry | 2012,2015,2020 | Presence of a registration system for opting-in and opting-out | Journal Article(s) | [Rosenblum (2012), Chua 2015](https://www.ncbi.nlm.nih.gov/pmc/articles/PMC3440579/), Interviews |
|  | Next-of-kin veto | 2011,2020 | Next-of-kin can veto donation decision | Journal Article(s) | [Rosenblum (2011)](https://www.ncbi.nlm.nih.gov/pmc/articles/PMC3363979/), Interviews |
|  | In-hospital Donor Coordinator | 2019 | Presence of an in-hospital donor coordinator (Yes/No) | Journal Article(s), personal interviews | Interviews |
|  | Role of Donor Coordinator | 2019 | Who takes on the role of donor coordinator | Journal Article(s), personal interviews |  |
|  | Brain death Legislation | 2015 or later | Whether the country has an official document for brain death legislation | Journal Article(s) | [Wahlster (2015)](https://www.ncbi.nlm.nih.gov/pmc/articles/PMC4433464/)  Chua (2015)  Citerio (2015)  Interviews |
|  | Brain Death diagnosis | 2015 or later | Who has the right to diagnose brain death in each country | Journal Article(s) |  |
