# Supplementary material for: Healthcare System Impact on Deceased Organ Donation and Transplantation: A Comparison Between the Top 10 Organ Donor Countries With 4 Countries in Southeast Asia
Source: Transpl Int. 2023 Aug 30;36:11233. doi: 10.3389/ti.2023.11233 (PMC10498995; doi:10.3389/ti.2023.11233)
Supplement: Supplementary file 2 [file Table2.docx]

**Supplementary table 2: Healthcare resources between countries**

Southeast-Asia

|  | F. Healthcare Resources | | | | | | |
| --- | --- | --- | --- | --- | --- | --- | --- |
|  | Physicians (per 1,000 population) | Surgical Workforce (pmp) | Neurosurgeons (per 10,000) | Nurses and Midwives (per 1,000 population) | Hospital Beds  (per 1,000 population) | ICU beds (per 100,000 population) | Transplant Centres  (# /per 100,000) |
| Spain | 3.87 | 7.99 | 11.2 | 5.73 | 2.97 | 9.70 | 40 / 8.5 |
| United States | 2.61 | 5.47 | 16.1 | 14.55 | 2.87 | 25.80 | 232 /7.1 |
| Croatia | 3.00 | 8.12 | 13.7 | 8.12 | 5.54 | 14.70 | 4 / 9.8 |
| Portugal | 5.12 | 8.61 | 16.2 | 6.97 | 3.45 | 4.20 | 8 / 7.8 |
| France  Countries leading in organ donation | 3.27 | 5.86 | 6.7 | 11.47 | 5.91 | 16.30 | 47 / 7.0 |
| Belgium | 3.07 | 8.98 | 14.1 | 19.46 | 5.58 | 17.40 | 7 / 6.1 |
| Czechia | 4.12 | 12.45 | 8.0 | 8.40 | 6.62 | 11.60 | 7 / 6.6 |
| Finland | 3.81 | 7.63 | 8.6 | 14.74 | 3.61 | 5.40 | 1 / 1.8 |
| Belarus | 5.19 | 12.42 | 15.0 | 11.00 | 10.83 | - | 7 / 7.4 |
| Malta | 2.86 | 11.35 | 6.0* | 9.48 | 4.49 | - | 1 / 19.8 |
| MEAN | 3.69 | 8.89 | 11.5 | 10.99 | 5.19 | 13.14 | 35.40 / 8.2 |
| Thailand | 0.81 | 1.31 | 5.2 | 2.76 | 2.10 | 10.40 | 28 / 4.0 |
| Malaysia | 1.54 | 1.14 | 3.4 | 3.47 | 1.88 | 3.40 | 6 / 1.9 |
| Philippines | 0.60 | 0.96 | 1.1 | 4.94 | 0.99 | 2.20 | 18 / 1.7 |
| Myanmar | 0.68 | 0.24 | 0.2 | 1.00 | 1.04 | 0.62 | 9 / 1.7 |
| MEAN | 0.90 | 0.91 | 2.5 | 3.04 | 1.50 | 4.16 | 15.25 / 2.3 |
